# Prospective Optical Lattice Clocks in Neutral Atoms with Hyperfine Structure

**DOI:** 10.3390/atoms12030014

**Published:** 2024

**Authors:** Tobias Bothwell

**Affiliations:** Time and Frequency Division, National Institute of Standards and Technology, Boulder, CO 80305, USA

**Keywords:** optical lattice clocks, forbidden transitions, AC stark clock shifts

## Abstract

Optical lattice clocks combine the accuracy and stability required for next-generation frequency standards. At the heart of these clocks are carefully engineered optical lattices tuned to a wavelength where the differential AC Stark shift between ground and excited states vanishes—the so called ‘magic’ wavelength. To date, only alkaline-earth-like atoms utilizing clock transitions with total electronic angular momentum J=0 have successfully realized these magic wavelength optical lattices at the level necessary for state-of-the-art clock operation. In this article, we discuss two additional types of clock transitions utilizing states with J≠0, leveraging hyperfine structure to satisfy the necessary requirements for controlling lattice-induced light shifts. We propose realizing (i) clock transitions between same-parity clock states with total angular momentum F=0 and (ii) M1/E2 clock transitions between a state with F=0 and a second state with J=1/2, $m_{F}=0$. We present atomic species which fulfill these requirements before giving a detailed discussion of both manganese and copper, demonstrating how these transitions provide the necessary suppression of fine structure-induced vector and tensor lattice light shifts for clock operations. Such realization of alternative optical lattice clocks promises to provide a rich variety of new atomic species for neutral atom clock operation, with applications from many-body physics to searches for new physics.

## 1. Introduction

Modern atomic clocks based on neutral atoms tightly confined in carefully engineered optical lattices, so-called optical lattice clocks (OLCs) [1,2], are the leading platform for next-generation frequency metrology. OLCs based on Sr and Yb have both demonstrated fractional frequency inaccuracies at the low 10^−18^ level [3–5] and fractional frequency instabilities at the mid 10^−17^ level at 1 s [6,7]. Lab-based clocks test fundamental physics [8–10] while portable OLCs have provided stringent tests of general relativity [11]. The quantum control afforded by OLCs has been leveraged to study SU(N) symmetry [12], synthetic spin–orbit coupling [13], multi-orbital polaron models [14], and even engineer clocks free of atomic interaction-induced frequency shifts [15]. Despite such advances, these devices promise to continue to improve rapidly as intra-clock comparisons are now reaching fractional frequency uncertainties at the 21st digit, resolving the gravitational redshift-induced frequency shift within a single OLC [16].

Central to the precision and accuracy of OLCs based on neutral Sr and Yb is the doubly forbidden clock transition between ground ^1^S0 and excited ^3^P0 clock states. The use of states with total electronic angular momentum J=0 limits perturbations to scalar shifts, avoiding experimentally unstable vector and tensor shifts [17]. This enables the realization of the magic wavelength optical lattice, where the ground and excited clock states are equally perturbed by the strong trapping field required for the Doppler-free interrogation of the clock transition. Typical OLCs use trap depths with energy scales of 100 kHz, with residual differential shifts now approaching the mHz level [18–20]; while tmagic wavelength lattices with vector or tensor polarizabilities arising from fine structure are possible, experimental fluctuations result in fractional variations relative to the full trap depth of 100 kHz, limiting the use of such atoms in optical lattice clocks.

Despite these challenges, there is an increasing push for alternative OLC atoms which deviate from the alkaline-earth architecture. Motivations for this are both practical and of fundamental scientific motivation. First, Sr and Yb are both weakly perturbed by room-temperature black-body radiation (BBR) at several parts in 10^15^ [17], requiring careful thermal engineering and temperature measurement to characterize BBR-induced clock shifts [3,21–23]. Atoms based on BBR insensitive inner-shell transitions in erbium or thulium seek to address this concern at the cost of increased complexity in other systematic shifts [24,25]. Second, it would be of great impact to have the accuracy and precision of OLCs applied to optical transitions with enhanced sensitivities to fundamental physics or novel interactions. Clocks based on Cu, Ag, and Au have been proposed, due to their sensitivities, for the variations of the fine-structure constant and Lorentz symmetry [26,27]. Clocks based on magnetic dipole (M1) and electric quadrupole (E2) transitions could be leveraged for parity non-conservation measurements [28]. Recently titanium was proposed as a clock with new interactions produced by magnetic dipole–dipole interactions [29], providing a testbed for long-range interactions with narrow optical transitions.

**Figure 1.**
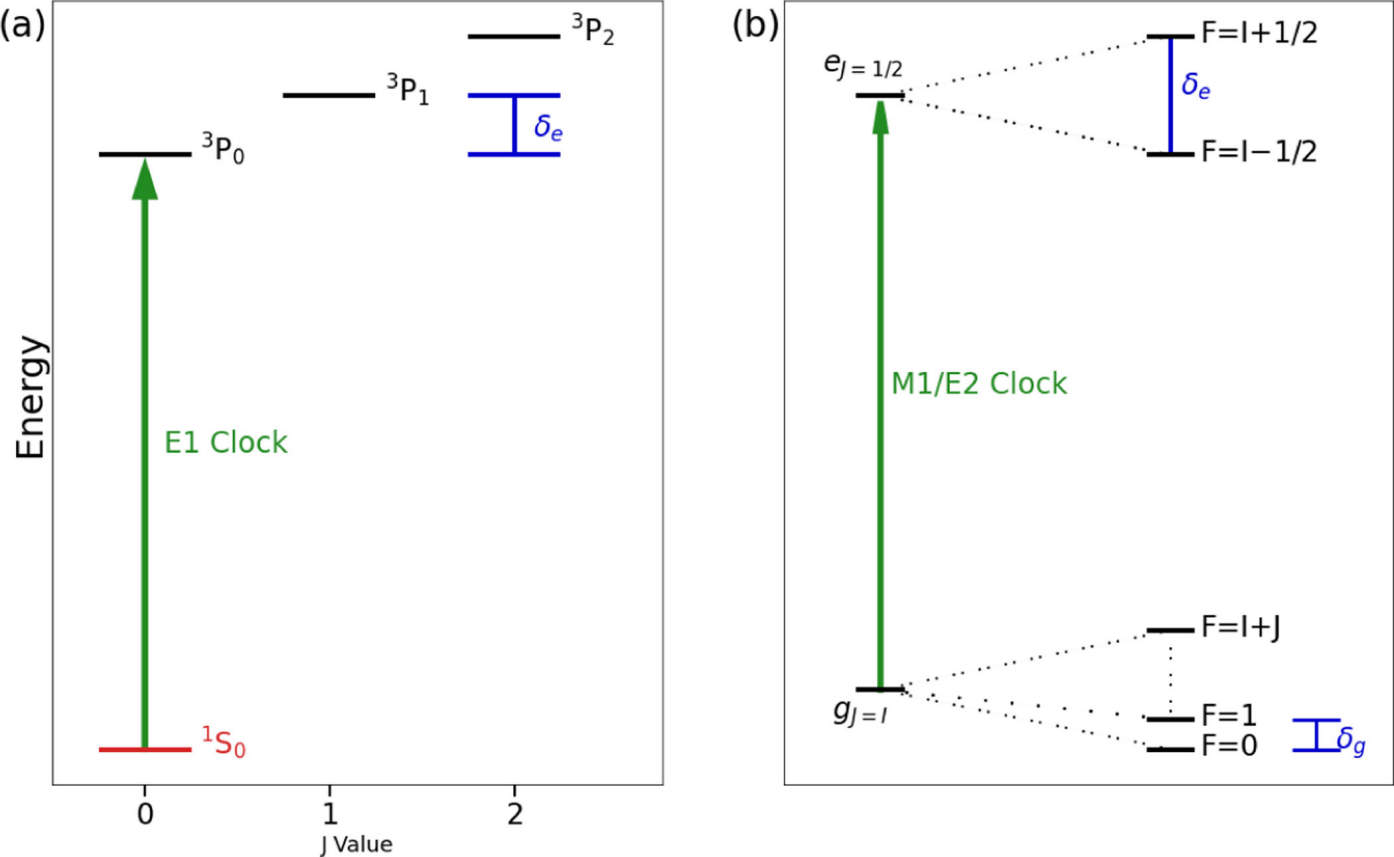
Viable clock transitions for controlling differential AC Stark shifts in clocks. (**a**) Standard alkaline-earth-like clock transition between states of an even (red) and odd (black) parity. The two states with the lowest energy are a doubly-forbidden E1 transition between ^1^S_0_ and ^3^P_0_. The energy difference between the excited clock state ^3^P_0_ and nearby ^3^P_1_ state, denoted $\delta_{e}$, sets the energy scale for typical higher-order perturbations. A related situation (not shown) is transitions between states of the same parity, both with F=0. In this case, the relevant energy splittings are given by the hyperfine splitting between F=0 and F=1. (**b**) M1/E2 clocks between a state with J=I and F=0 and a second state with J=1/2, F=1,2, and $m_{F}=0$ operate free of vector and tensor shifts. In this case, the energy splitting between nearby states is set by the hyperfine splitting, not fine structure splitting, enhancing higher-order perturbations. This scheme requires half-integer spins for both *J* and nuclear spin (I), with at least one state where J=I.

Inspired by these proposals, we suggest clock transitions which fulfill the necessary conditions for accurate light shift control. We propose exploring two new types of clock transitions in bosonic atomic species with hyperfine structure (Figure 1). (1) A M1/E2 clock can be operated between a state with total angular momentum F=0 and a second clock state with J=1/2, $m_{F}=0$. (2) Electric-field-induced or two-photon spectroscopy can be utilized to probe forbidden M1/E2 transitions between states with both the same parity and zero total angular momentum ($F=F^{\prime }=0$, where ^′^ denotes the upper clock state). Both types of transitions may lead to new OLC platforms, enhanced sensitivities to new physics, and novel explorations of many-body physics systems. As examples, we consider clock operation in both manganese (Mn) and copper (Cu), two unexplored species within the laser cooling community.

We organize the paper as follows. In Section 2, we review the AC Stark formalism necessary for characterizing and understanding lattice light shifts in optical lattice clocks. We emphasize how the lattice light shift control—as demonstrated in alkaline-earth-like atoms can be realized in the aforementioned schemes, with a discussion addressing new systematic considerations. Examples of transitions in various atomic species are given. In Section 3, we introduce neutral Mn, discussing the tools for laser cooling and preparation. Section 4 builds on this, providing an overview of the proposed clock transitions, showing multiple clock transitions within a single neutral atom. A short discussion on standard systematics is given, as well as new shifts, to address issues arising from the use of hyperfine states. Section 5 follows from the previous two sections, applying our findings to a second species of interest—Cu. Section 6 concludes by briefly summarizing our findings.

## 2. Lattice Light Shifts in Optical Lattice Clocks

We begin with a brief review of the differential AC Stark induced frequency shifts in OLCs, the so called lattice light shift systematic [17]. We note how deviation from $J=0↔J^{\prime }=0$ clock transitions creates a new set of couplings for frequency perturbations. With these considerations in mind, we then review the more general AC Stark formalism and detail viable architectures consistent with clock level control of lattice light shifts.

### 2.1. An Introduction to AC Stark-Induced Frequency Shifts in OLCs

We first consider the idealized case of a 1D OLC utilizing $J=0↔J^{\prime }=0\;$ transitions. The AC Stark shift for the ground (g) or exited state (e) is given by

(1) $$hv_{g(e)}=-\frac{1}{4}\alpha_{g(e)}^{S}(\omega )|E{|}^{2}$$

where $v_{g(e)}$ denotes the AC Stark shift in units of Hertz to either the ground or excited state, $\alpha_{g(e)}^{S}(\omega )$ is the corresponding scalar dipole polarizability resulting from the radiation of frequency ω, and E characterizes the amplitude of the radiation’s electric field. Once more, $v_{g(e)}$ is of order 100 kHz, corresponding to a clock shift at the 1 × 10^−10^ level. The idea of the ‘magic’ wavelength is to minimize the differential AC Stark shift between clock states, $\text{Δ}v_{LS}$, as given by

(2) $$\begin{array}{l} h\text{Δ}v_{LS}(\omega )=-\frac{1}{4}\left(\alpha_{e}^{S}(\omega )-\alpha_{g}^{S}(\omega )\right)|E{|}^{2}\\ \;=-\frac{1}{4}\text{Δ}\alpha^{S}(\omega )|E{|}^{2}, \end{array}$$

(3) $$\left|h\text{Δ}v_{LS}\right|\;\ll 100\;\text{kHz}.$$

Here, $\text{Δ}\alpha^{S}(\omega )$ denotes the differential scalar polarizability between clock states. When the lattice wavelength is tuned to minimize $\text{Δ}v_{LS}$, the trapping conditions are an excellent approximation identical for both states, critical for decoupling electronic from motional degrees of freedom. In practice, the systematic lattice light shift in OLCs is the study of residual perturbations of Equation (3), with current research at the mHz scale. Complications leading to shifts at this level include higher-order polarizability effects, characterization of motional states, and measurement and stability of trap depth. Absent from Equation (3) is vector or tensor contributions, arising from the use of J≠0 states, which is discussed below.

We may contrast this with a more general treatment of the lattice light shift where we include vector and tensor terms. Assuming *π*-transitions, the differential clock polarizability is often written as in Refs. [30,31]:

(4) $$\text{Δ}\alpha (\omega )=\text{Δ}\alpha^{S}(\omega )+\xi \cos\left(\theta_{k}\right)\frac{m_{F}}{2F}\text{Δ}\alpha^{V}(\omega )\;\;+\left(\frac{3\cos^{2}\left(\theta_{p}\right)-1}{2}\right)\frac{3m_{F}^{2}-F(F+1)}{F(2F-1)}\text{Δ}\alpha^{T}(\omega )$$

(5) ≈0

where $\text{Δ}\alpha^{S}(\omega )$, $\text{Δ}\alpha^{V}(\omega )$, and $\text{Δ}\alpha^{T}(\omega )$ represent the differential scalar, vector, and tensor polarizability of atoms exposed to the radiation of frequency ω. ξ is the degree of circular polarization of the lattice light polarization (0 for linear, 1 for circular), $\theta_{k}$ is the angle between the lattice and quantization axes, $\theta_{p}$ is the angle between the polarization and quantization axes, F is the total angular momentum quantum number, and $m_{F}$ is the corresponding projection. Note that this treatment assumes a well-defined magnetic quantization axis that is not significantly perturbed by a large vector AC Stark shift.

The reduced dipole matrix elements for all three polarizability contributions in Equation (4) are the same. Without exploiting specific angular momentum states (J=0 or $m_{F}=0$, for example) there is no a priori reason for the vector or tensor polarizabilities to be significantly smaller than the scalar polarizability [29]. A magic wavelength operation in such regimes requires an operational point where Equation (5) holds. Critically, this does not ensure that $\text{Δ}\alpha^{S}(\omega )=\text{Δ}\alpha^{V}(\omega )=\text{Δ}\alpha^{T}(\omega )=0$. Thus, while a magic wavelength with J≠0 states is, in principle, possible, experimental fluctuations may result in fractional changes relative to the 100 kHz energy scale of the optical lattice depth.

This may be compared with F>0, J=0 clock states, which exhibit deeply suppressed scalar and tensor shifts. Consider ^87^Sr clocks, operating on the standard J=0 clock states. ^87^Sr is a fermionic isotope, which, from the state mixing induced by the nuclear spin I=9/2, has a mHz linewidth transition. This mixing weakly introduces vector and tensor polarizabilities, originally estimated to be suppressed at a level of the electron-to-proton mass (≈10^−4^) relative to the scalar polarizability [32]. While this estimate holds for the vector polarizabilities, the tensor polarizabilities are further suppressed due to angular momentum rules; in ^87^Sr they are suppressed by ≈10^−6^ relative to the scalar polarizability [31]. Trapping conditions are, thus, well described by the scalar dipole polarizability, with trap depths effectively independent of the vector and tensor contributions. As the vector shifts do not perturb the trapping conditions (motional wavefunctions), Sr clocks are able to average between opposite $m_{F}$ projections to eliminate vector shifts. The residual tensor contribution requires more care. It is standard practice to align $\theta_{p}$ to be either 0 or π/2, ensuring tensor shifts are first-order-insensitive to small fluctuations in the angle. Such alignment at the 1° level is commonplace, limiting the instability of the tensor lattice light shift to the relative level of 10^−4^. Combining this with the suppression of the tensor shift from the J=0 nature of the clock leads to the instability of tensor-induced shifts in Sr clocks at a level of 10^−10^ relative to the trap depth. A similar effect exists for states with J=1/2 where tensor shifts are eliminated in the case of I=0 or weekly admixed as discussed for I>0, F>0.

In contrast, a clock with fine structure level differential vector and tensor polarizability must confront far larger shifts. In such a clock, a drift in $\theta_{p}$ from 0° to 0.5° would result in an approximately 20 Hz shift, a fractional frequency change in the Sr clock transition of 5 × 10^−14^; while there may exist magic wavelengths with inherently smaller tensor contributions, suppression at the level of 10^5^ is unlikely. Similarly, a full-scale vector shift would not only lead to such frequency shifts, but would fundamentally be incompatible with vector-averaging techniques as the trap depth would become strongly *m*_*F*_-dependent. In short, the implementation of magic wavelengths for clock performance breaks down in J≠0 states, with many of the alternative atomic species discussed in the introduction likely unable to reach 10^−18^ level clock accuracy.

### 2.2. Full AC Stark Treatment for Lattice Light Shifts

Alternative clocks would benefit from transitions free of vector and tensor shifts. For a more careful treatment of the AC Stark shift, we follow [33], building on Equation (4). Consider the scalar, vector, and tensor polarizabilities for state *nJF* subjected to a given radiation frequency. The corresponding polarizabilities, in atomic units, are given by

(6) $$\alpha_{nJF}^{S}=\alpha_{nJ}^{S}=\frac{1}{\sqrt{3(2J+1)}}\alpha_{nJ}^{\left(0\right)},$$

(7) $$\alpha_{nJF}^{V}={\left(-1\right)}^{J+I+F}\sqrt{\frac{2F(2F+1)}{F+1}}\left\{\begin{array}{lll} F & 1 & F\\ J & I & J \end{array}\right\}\alpha_{nJ}^{\left(1\right)},$$

(8) $$\begin{array}{l} \alpha_{nJF}^{T}=-{\left(-1\right)}^{J+I+F}\sqrt{\frac{2F(2F-1)(2F+1)}{3(F+1)(2F+3)}}\\ \;\times \left\{\begin{array}{ccc} F & 2 & F\\ J & I & J \end{array}\right\}\alpha_{nJ}^{\left(2\right)}, \end{array}$$

where fine structure polarizabilities are given by

(9) αnJ(K)=(−1)K+J+12K+1×∑n′J′(−1)J′1K1JJ′Jn′J′‖d‖nJ2×1ℏRe1ωn′J′nJ−ω−iγn′J′nJ/2+(−1)Kωn′J′nJ+ω+iγn′J′nJ/2.

Here, $\omega_{n^{\prime }J^{\prime }nJ}$ corresponds to the angular frequency between states nJ and $n^{\prime }J^{\prime }$ with linewidth $\gamma_{n^{\prime }J^{\prime }nJ}$. $\left|\langle n^{\prime }J^{\prime }|\right|d||nJ\rangle {|}^{2}$ is the corresponding magnitude squared reduced electric dipole matrix element between the clock state and relevant opposite parity state, and the bracketed terms are Wigner 6-j symbols. This framework allows us to identify $J\neq 0↔J^{\prime }\neq 0$ transitions which follow the established alkaline-earth recipe of eliminating or deeply suppressing non-scalar lattice light shifts.

### 2.3. Spectroscopy of Forbidden F=0 to $F^{\prime }=0$ Transitions of States with the Same Parity

As discussed, alkaline-earth-like atoms operate on the doubly forbidden ^1^${\text{S}}_{0}{↔}^{3}{\text{P}}_{0}$ clock transitions. The clock spectroscopy of such forbidden transitions requires state mixing between ^3^P_0_ and the nearby ^3^P_1_ state via either the hyperfine interaction of isotopes with a nuclear spin or the application of a large magnetic field, which mixes states of the same parity [34]. We note that in addition to $J=0↔J^{\prime }=0$ transitions between opposite parity states, there exist such transitions between states of the same parity [34–36].

Utilizing hyperfine structure, it is clear from Section 2.2 that F=0 states are free of vector and tensor shifts. We, thus, identify transitions between F=0 states with the same parity as a viable clock transition compatible with accuracy. In analogy to magnetic-field-induced spectroscopy, theses transitions may be driven by an admixture of opposite parity states via the application of a DC electric field.

In addition to electric-field-induced spectroscopy, the proposed transitions may be excited by two-photon spectroscopy. Similar schemes for the alkaline-earth-like architecture have been proposed using an E1-M1 photon pair [37,38]. In the case of transitions between states with the same parity, the two-photon scheme becomes an E1-E1 excitation, strongly reducing the probe-induced light shifts. Such a two-photon transition could be useful for suppressing Doppler-shifts present in emerging tweezer clock platforms [39] or in enabling new atoms for compact, two-photon clocks [40].

Examples of possible clock transitions are given in Table 1. A discussion on the implementation is presented in Section 4.2.1.

### 2.4. Spectroscopy between Same-Parity States with F=0, J=1/2, $m_{F}=0$, and F=1,2

In parallel to forbidden transitions between states with F=0 and the same parity, there exist specific M1/E2 clock transitions that satisfy our stringent lattice light shift constraints. Consider one clock state with F=0 and a second clock state with J=1/2, F=1,2, and $m_{F}=0$. From Equation (9), we see that for states with J=1/2, the Wigner 6-j symbol is zero, eliminating tensor shifts (higher-order contributions at the level of 10^−4^ will exist as discussed previously). Utilizing $m_{F}=0$, the state is also free of vector shifts. We, thus, find that there are additional M1/E2 clock transitions which fulfill the ideal requirements of vector and tensor shift-free magic wavelength trapping.

Limiting ourselves to M1/E2 transitions, finding such clocks amounts to a search of the periodic table for atoms with an odd number of protons and nuclear spin I=1/2,3/2,5/2. Examples are given in Table 2.

### 2.5. New Systematics

The proposed clock transitions are not without complication, with the hyperfine structure of J≠0 states resulting in new systematic considerations. The energy scale of second-order shifts (Zeeman and AC Stark) in alkaline-earth-like clocks is set by the energy splitting between the excited clock state ^3^*P*_0_ and nearby ^3^*P*_1_ state [17], typically of the order of 10 THz. For hyperfine states, this scale is 1 GHz, resulting in the enhancement of second-order shifts. Figure 1 illustrates this difference.

#### 2.5.1. Second-Order Zeeman Shift

First- and second-order Zeeman shifts in alkaline-earth-like clocks are deeply suppressed owing to the $J=0↔J^{\prime }=0$ nature of the clock transitions [17]. In contrast, the second-order Zeeman shift in the proposed clock transitions is set according to Ref. [41]:

(10) $$\Delta v_{Z2}\approx \frac{{\left(\mu_{B}B\right)}^{2}}{\Delta_{HF}}$$

where $\mu_{B}$ is the Bohr magneton (1.4 MHz/G), B is the magnetic field amplitude, and $\Delta_{HF}$ is the hyperfine scale energy splitting of an order of 1 GHz. While potentially large, the use of hyperfine states with either F=0 or $m_{F}=0$ means that mG-level fields are sufficient to resolve mF states in contrast to the G-level fields used in current clocks, enabling a strong suppression of the second-order Zeeman shift. Further, an applied magnetic field could be readily measured and actively stabilized via magnetically sensitive clock transitions to $m_{F}\neq 0$ states.

#### 2.5.2. Hyperpolarizability

Similar to the second-order Zeeman shifts, the fourth-order light field effects (hyperpolarizability) are deeply suppressed in standard OLCs. The use of hyperfine structure of J≠0 states leads to significantly larger hyperpolarizability. The scale of hyperpolarizability is set according to Ref. [42]

(11) $$\Delta v_{H}\approx \frac{U^{2}}{\Delta_{HF}}$$

where U is the associated trap depth. For typical trap depths like U=100 kHz, this shift corresponds to order 10 Hz. With cooling and careful preparation, operational depths can be 10 times smaller, pushing such shifts into the sub-Hertz regime. However, this is still far from the mHz shifts for 10^−18^ accuracy.

To address this large shift, an experiment may use a trap depth-dependent magic wavelength where the E1 polarizability is adjusted to offset the hyperpolarizabilty contribution at a specific trap depth. A local operational regime may be defined by

(12) $$\Delta v_{AC}\approx \Delta \alpha_{E1}\left(\omega_{L}\right)U+\beta U^{2}\approx 0,$$

where factors accounting for motional distributions and temperature have been neglected. Here, $\Delta v_{AC}$ is the residual differential AC Stark shift, $\Delta \alpha_{E1}\left(\omega_{L}\right)$ is the differential E1 polarizability for the radiation of frequency $\omega_{L}$, and β is the hyperpolarizability coefficient. The clock may then be operated at an operational depth $U_{op}$ where the E1 polarizability is tuned to give either $\Delta v_{AC}\approx 0$ or $\partial \Delta v_{AC}/\partial U\approx 0$ [18,19], dependent on the leading uncertainty within the light shift evaluation.

## 3. Neutral Manganese: Atomic Properties and Laser Cooling

To solidify the discussion of the proposed clock transitions, we first focus on the case study of neutral manganese. Neutral manganese is a highly magnetic atom that offers an abundance of clock transitions fitting both proposed strategies, offering unique multi-clock operational capabilities in a single atom, while other atoms in Table 2 may prove better for pure accuracy purposes, Mn, nonetheless, emphasizes the appeal of the proposed clock transitions.

Manganese, with atomic number Z = 25, is a transition metal appearing in the fourth row of the periodic table. It possesses a single stable isotope (^55^Mn) with a nuclear spin I=5/2 and ground state electronic configuration of 3d^5^4s^2^. The vapor pressure of Mn offers an oven operation near 1000 °C, significantly cooler than other laser-cooled atoms (Er, Dy, Ho, and Cr). Manganese is affordable and readily available in metallic form.

**Figure 2.**
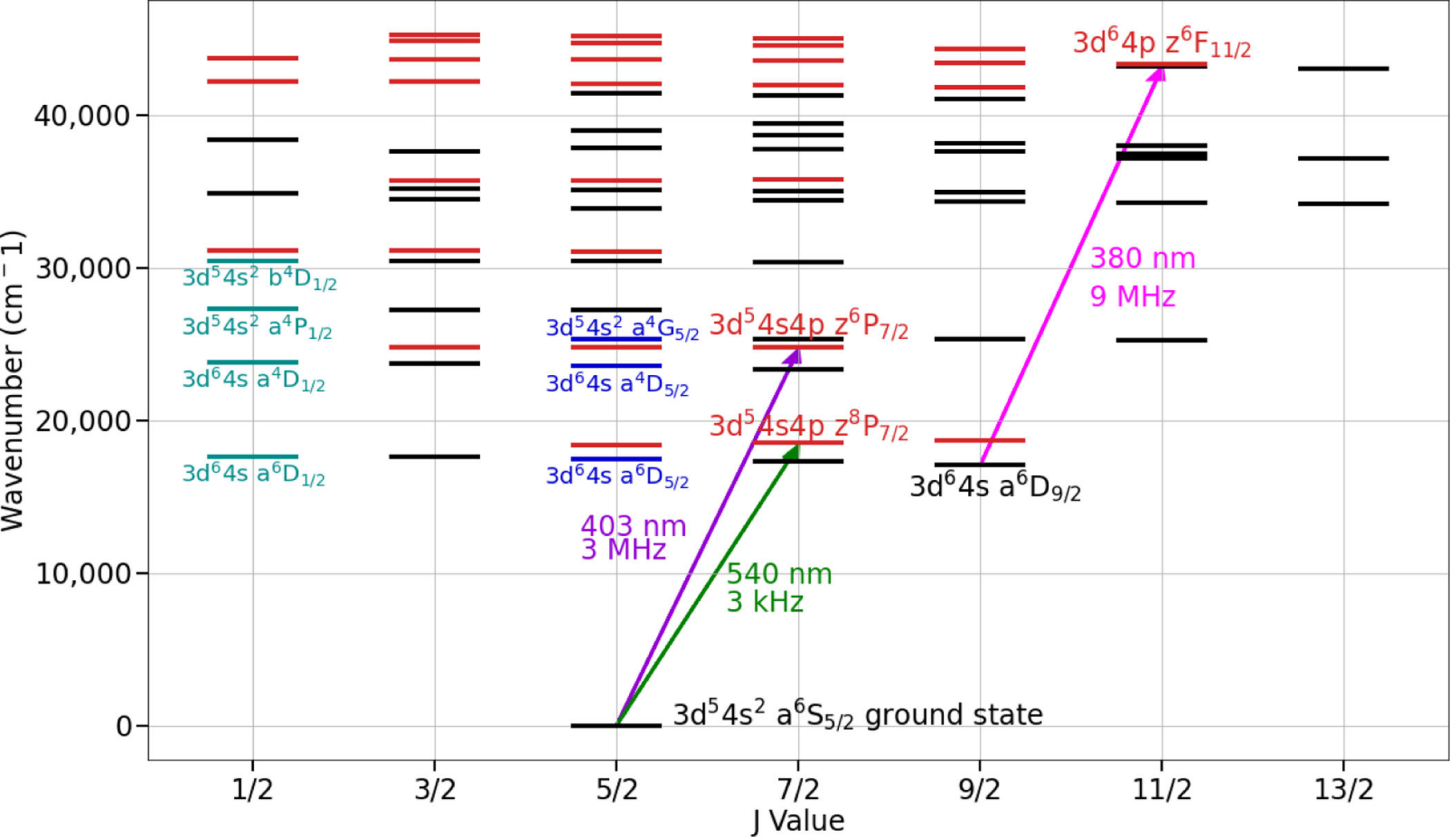
Mn energy level structure with odd parity states in black and even in red. States of interest for laser cooling and clock operation are labeled next to their respective lines. Clock transitions for $F=F^{\prime }=0$ are are in dark blue while clock transitions to J=1/2, $F^{\prime }=2$, mF=0 states are in dark turquoise. The ionization limit is 60,000 cm^−1^.

The atomic structure of Mn presents a rich selection of states as shown in Figure 2. At low energy levels, Mn is dominated by a spectrum of long lived odd parity states. The light atomic mass of Mn ensures that LS coupling remains a good description, largely suppressing spin flipping transitions prevalent in heavier atoms. Despite the relative complexity, the presence of a single isolated ground state with an effectively closed narrow-line transition enables a precise manipulation of hyperfine states.

### 3.1. Oscillator Strengths and Lifetimes

A discussion on neutral Mn requires oscillator strengths and lifetimes for clock states. For complicated atomic spectra, a leading technique is the use of semiclassical atomic structure calculations with least squares fitting to known atomic lines. The workhorse technique for this remains the so-called ‘Cowan-code’ [43,44]. A database of such fits for numerous atoms and ions with a proton number under 50 is available [45], providing us with oscillator strengths and lifetimes for Mn atomic states. We emphasize that many atoms in Table 2 are found in the database.

The Kurucz database [45] provides weighted oscillator strengths gf, which are defined by Ref. [43]

(13) $$gf=\left(2J_{i}+1\right)f_{ij}$$

where $J_{i}$ is the total electronic angular momentum of the lower level i, with j denoting the upper level. We may convert weighted electric dipole oscillator strengths to Einstein A coefficients $\left(A_{ij}\right)$ as in Ref. [43]:

(14) $$A_{ji}=\frac{1}{2J_{j}+1}\frac{6.6702\times 10^{-5}gf}{\lambda^{2}}s^{-1}$$

where λ is the wavelength in meters and $J_{j}$ is the total angular momentum of the upper level j.

### 3.2. Metastable MOT Operation

The standard tool for capturing and cooling atoms to a high phase space density is the magneto-optical trap (MOT) [46]. MOT operation requires the ability to scatter many photons without loss to another state. If such decays exist, atoms in the dark states must be pumped back into the cooling cycle. We may quantify the ratio of photons scattered to leakage (R, inverse of the branching ratio) as

(15) $$\frac{1}{R}=1-\frac{\sum_{b}A_{b}}{\sum_{a}A_{a}}$$

where *b* represents states which are scattered by the MOT or repumped, and *a* is summed over all states that the upper state may decay to. Laser cooling is typically effective with *R* > 10,000.

MOT operation from the ground state a^6^S_5/2_ (3d^5^4s^2^) to the upper state z^6^P_7/2_ (3d^5^4s4p) has been suggested [47,48]. However, the upper state has a large probability to flip a p orbital to d, leading to a rather large branching ratio to the a^6^D manifold (Figure 2). The Einstein A coefficients for various decays of the upper level are given in Table 3. We see that for the 403 nm transition, the state decays elsewhere after ≈30 photons are scattered. Repumping is impractical and hindered by complex dark states [49].

We instead explore the MOT operation on the metastable state a^6^*D*_9/2_, populated using the aforementioned 403 nm transition. We propose operating the MOT on the 381 nm transition to the upper MOT state z^6^*F*_11/2_. With a linewidth of ≈9 MHz, the transition is well suited to laser-cooling atoms from an atomic beam source. Furthermore, the wavelength is conveniently within range of commercial laser diodes.

Table 4 lists the decay pathways from the excited state z^6^*F*_11/2_. We find that, for the 381 nm transition, the MOT is relatively closed without any repump lasers (R ≈ 20,000). The addition of a single repump from a^4^G_11/2_ to y^6^F_11/2_ at 439 nm improves this to R ≈ 300,000, which is sufficient for the MOT operation. Atoms may be pumped to the ground state using a laser at 1290 nm (Table 3).

Sub-Doppler Cooling and a Novel Continuous Cold Atom Source

A full consideration of the hyperfine structure of Mn and its impact on MOT operation is beyond the scope of this paper. However, we note that for a standard MOT, the ground MOT state will operate on the F=7 state while the excited state will operate on F=8. The ground (excited) state Lande-g factor $\left(g_{J}\right)$ is 1.559 (1.464). For the MOT states, we may, thus, find the hyperfine Lande-g factor $\left(g_{F}\right)$ as

(16) $$g_{F}=g_{J}\frac{F(F+1)-I(I+1)+J(J+1)}{2F(F+1)}$$

where I is the nuclear spin quantum number, J the electronic angular momentum number, and F the total angular momentum number. We find that *g*_*F*_ for the ground (excited) MOT state is 1.002 (1.007), nearly degenerate at a level of 0.5%. Such nearly degenerate Lande-g factors enable efficient sub-Doppler cooling within standard MOT operation [50]. Critically, Mn has a nearly equal degeneracy of Lande-g factors (and linewidth) as in thulium, where sub-Doppler cooling in the MOT led to atomic temperatures of 25 μK [51].

Direct laser cooling to 25 μK in a MOT operated on a metastable state may provide a novel continuous atomic source. As the lifetime of the metastable MOT ground state is ≈1 s, continuously operating a 3D MOT on 381 nm will provide a steady supply of cooled atoms via decay from a^6^D_9/2_, *F* = 7 to a^6^S_5/2_, *F* = 5 where the atoms may be readily outcoupled. Owing to the deep sub-Doppler cooling which should be present in the MOT, such atoms may be directly loaded into an optical trap where they can be either further cooled or pumped to *F* = 0 via the narrow linewidth transitions available from the Mn ground state (Figure 2). Such continuous trapping of atoms from a single MOT stage that is decoupled from other atomic states of interest would be a vital resource with applications from active clocks [52–54] to continuous atom lasers [55].

### 3.3. Narrow-Line MOT

From the ground state of Mn, a series of weak intercombination lines is present near 540 nm. The lines, with kHz linewidths, are too weak to use without first cooling atoms in the first stage of MOT at 381 nm. However, once atoms are cooled by the first MOT stage, they may be further cooled and prepared into the desired initial state.

We consider the 540 nm transition from a^6^*S*_5/2_ to z^8^*P*_7/2_. The transition has a linewidth of 3 kHz. Critically, the transition is effectively closed (*R* ≈ 10,000) for a narrow transition (Table 5). This ensures the preparation of recoil-limited 1 μK samples and efficient state preparation, borrowing heavily from the established methods of alkaline-earth-like atoms.

## 4. Mn Clock Transitions

As shown in Figure 2 and listed in Table 6, Mn possesses both types of the newly proposed clock transitions. This section briefly reviews these Mn clock transitions before discussing systematics.

### 4.1. Allowed E2 Transitions

Transitions from |g, F=0〉 to |e, F=2, mF=0〉 are allowed via E2 selection rules and fulfill the AC Stark requirements set out at the beginning of this paper. Mn possesses several such transitions (Table 6). We briefly note that magnetic dipole–dipole interactions (DDIs) will introduce possible new systematics which may be readily accounted for as in Ref. [29]. However, the proposed F=2 excited states have smaller Lande-g factors than those discussed for Ti transitions [29] and can be further mitigated by long-wavelength optical lattices. Furthermore, ^4^*D*_1/2_ states are, under LS coupling, magnetically insensitive, ensuring viable clock transitions free of magnetic DDIs in Mn.

### 4.2. Forbidden M1/E2 Transitions

Utilizing F=0 states ensures no first-order magnetic sensitivity and removes magnetic DDI sytematics. However, such transitions are forbidden and require additional considerations to coherently interrogate.

#### 4.2.1. Electric-Field-Induced Forbidden E2 Transitions

As discussed, one method for enabling clock interrogation of forbidden transitions between states of the same parity is to apply an electric field, mixing states of opposite parity and enabling a weak admixture of an E1-allowed decay channel. We consider an excited clock state |e〉 and nearby states of opposite parities (|n〉) separated in energy by $\Delta_{e,n}$. Upon the addition of a static electric field $\left(\vec{E}\right)$, the states become coupled by matrix element Ω_*E*_, resulting in a dressed state

(17) $$|e^{\prime }\rangle =|e\rangle +\frac{\Omega_{E}}{\Delta_{e,n}}|n\rangle .$$

A full estimate of this effect for states in Table 6 is beyond the scope of this paper. We instead note that complimentary magnetically induced clock transitions are an established technique [34].

#### 4.2.2. Two-Photon Transitions

We additionally note that as these transitions are between states of the same parity, a standard two-photon excitation scheme may be used. Such techniques have been proposed in alkaline-earth-like level structures [37,38]. Here, the transition is driven by two E1 couplings, leading to an enhancement of the transition strength by 1/*α*_*FS*_ and a corresponding reduction in the probe-induced AC Stark shift of nearly 10^5^, a major improvement over two-photon (E1/M1) transitions between states of different parities.

### 4.3. Blackbody Radiation-Induced Frequency Shifts

The largest perturbation and a limiting system for state-of-the-art OLCs is the blackbody radiation shift [17]. Enticingly, transition metals have lower static electric polarizabilities than alklaline-earth atoms and, correspondingly, smaller BBR shifts. The use of the same parity states for the clock provides additional opportunities for cancellation of differential polarizabilities.

For the leading order, the BBR shift induced by the differential static electric dipole polarizabilities between the clock states $\left(\Delta \alpha_{0}\right)$ is given by Ref. [56]:

(18) $$\Delta v_{BBR}=-\frac{1}{2}{\left(831.9\;\text{V}/\text{m}\right)}^{2}{\left(\frac{T(K)}{300}\right)}^{4}\Delta \alpha_{0},$$

where T(K) is the temperature of the BBR in K. The static electric–dipole polarizabilitiy is readily found (in atomic units) as in Ref. [57]:

(19) $$\alpha_{0}=\sum_{j}\frac{f_{ij}}{{\left(\Delta E_{ij}\right)}^{2}}$$

where both equations are in atomic units, with $\Delta E_{ij}$ being the difference in energy between the state of interest i and all E1-connected states j. Using the oscillator strengths discussed in Section 3.1, we evaluate the BBR shifts for the clock states, with anticipated clock sensitivities given in Table 6. We find several transitions with BBR shifts nearly a factor of 20 (50) lower than in Yb (Sr), comparable to challenging UV atoms like Hg and Cd and consistent with the BBR shifts of other transition metals (Cu,Ag,Au) [26]. Such reduction in BBR shifts provides a compelling motivation for the exploration of transition metal-based OLCs.

### 4.4. Magic Wavelengths

**Figure 3.**
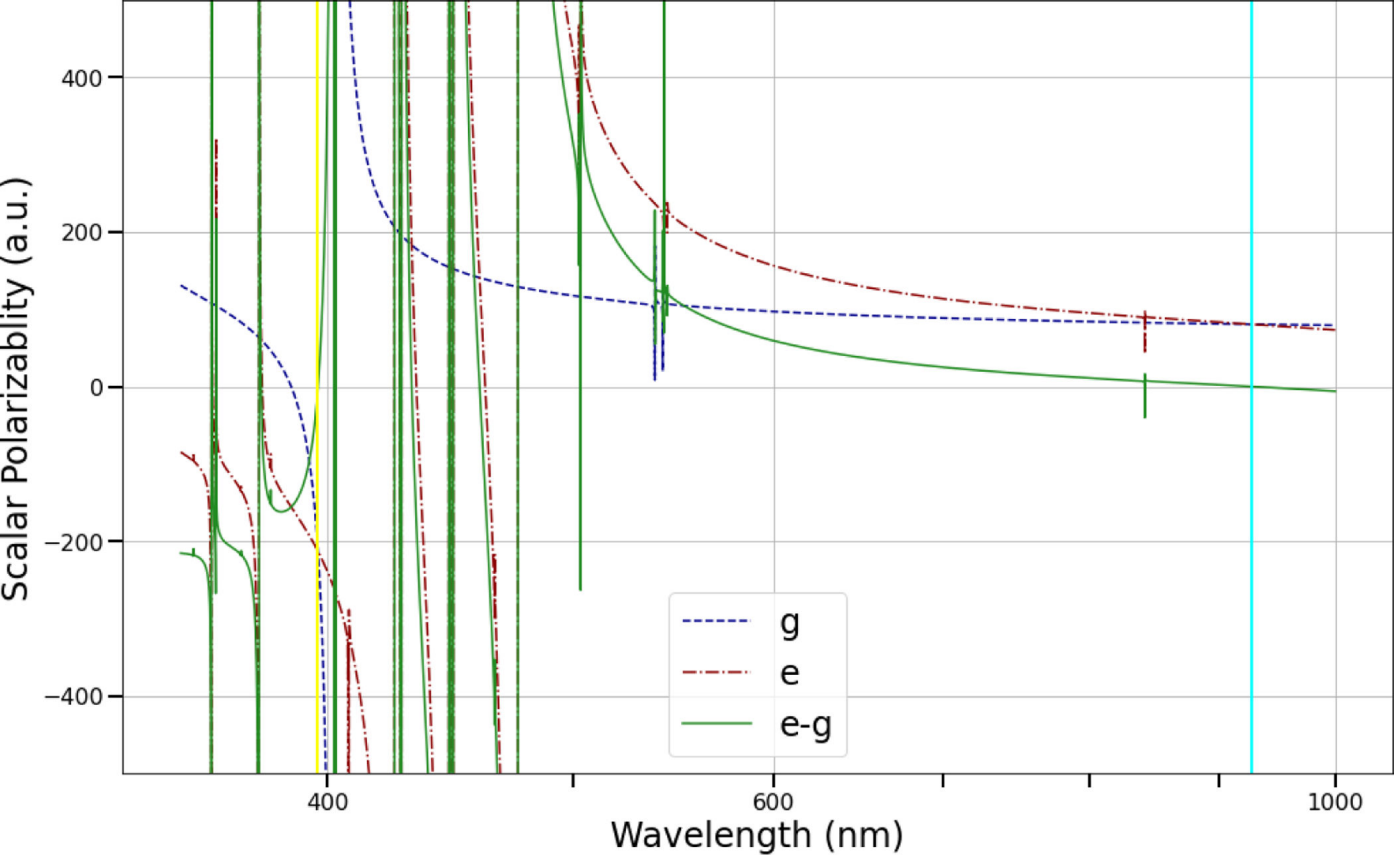
Scalar polarizabilities as a function of lattice wavelength for the 420 nm transition in Mn. Numerous magic wavelengths exist, with a red-detuned wavelength near 927 nm (cyan line) and blue-detuned wavelength near 396 nm (yellow line) providing novel options for both clock and magnetic DDI tuning.

Predicting magic wavelengths using atomic structure calculations of the least-squares fitting of atomic levels is notoriously imprecise in comparison to calculating the DC polarizabilities. Nonetheless, the rich structure of Mn ensures multiple magic wavelengths will be readily available for the clock transitions proposed. In general, for states with multiple E1 transitions, general magic wavelength conditions may be found. We emphasize this by focusing on the case of the 420 nm clock transition. The scalar E1 polarizability with wavelength dependence is given by

(20) $$\alpha_{0}(\omega )=\sum_{j}\frac{f_{ij}}{\Delta E_{ij}^{2}-\omega^{2}}$$

where ω is the energy of the corresponding frequency of radiation (atomic units are, once more, used). From our oscillator strengths, we can, thus, calculate the frequency-dependent scalar polarizability for the 420 nm transition, as shown in Figure 3. Owing to the infrared transitions between the a^4^D and z^6^P manifolds, there will exist a red detuned magic wavelength far detuned from any strong dipole lines (near 927 nm). Similarly, there will be a strong polarizability, blue-detuned magic wavelength near 396 nm. Numerous other magic wavelengths exist in the 300 nm to 450 nm range, ensuring a variety of viable options for the Mn clock operation.

### 4.5. New Systematics and Their Mitigation

As discussed, the use of hyperfine states in the J≠0 clock states leads to new systematics. Here, we present estimates of the magnitude of the effects in Mn and approaches to mitigation.

### 4.6. Second-Order Order Zeeman Shift

In Mn, the second-order Zeeman shift will be dominated by the ground state due to its small hyperfine splitting $\left(\Delta_{HF}=72\text{MHz}\right)$ [58]. From Equation (10), we find a shift of nearly 30 kHz/G^2^, meaning the Mn clock operation will require minimizing or stabilizing magnetic fields. At low magnetic fields, the degeneracy of nearby *m*_*F*_ states may be readily lifted by a weak admixture of circular polarization on a lattice beam, utilizing an effective magnetic field to set the quantization axis. An operation with 100 μG fields (and corresponding uncertainty) will enable a clock inaccuracy at 1 × 10^−18^. Field zeroing and stability at the 10 μG level have been demonstrated in standard atomic physics machines [59], which would push this shift and uncertainty to the 10^−20^ level.

### 4.7. Second-Order AC Stark–Hyperpolarizability

In Section 2.5.2, we discuss the enhancement of second-order AC Stark shifts in the clock. For trap depths of order 100 kHz and the hyperfine splitting of $\Delta E_{HF}$, we find

(21) $$\begin{array}{l} \Delta v_{H}(U)=\frac{{\left(100\;\text{kHz}\right)}^{2}}{72\;\text{MHz}},\\ \;\approx 140\;\text{Hz}. \end{array}$$

Thus, in Mn, there exists a strong hyperpolarizability effect. Reducing trap depths to ≈20 kHz brings this shift down to ≈5 Hz scales. This presents the largest shift in the proposed Mn clock.

Following Section 2.5.2, one approach is to set the first-order AC Stark shift to cancel the hyperpolarizabliity contribution for a specific trap depth, resulting in zero lattice light shifts to the first order. This provides a powerful method of detuning lattice beams in a *>*1D optical lattice clock without creating frequency shifts throughout the atomic sample. With a trap depth stabilization of .5%, this corresponds to a fractional frequency inaccuracy of 1 × 10^−17^. Accuracy at the eighteenth decade and beyond will require active feedback of trap depth via interleaved measurements of the trap depth-induced light shifts. While cumbersome, similar techniques are already employed in state-of-the-art clocks [60].

## 5. Prospects for a Copper Clock

**Figure 4.**
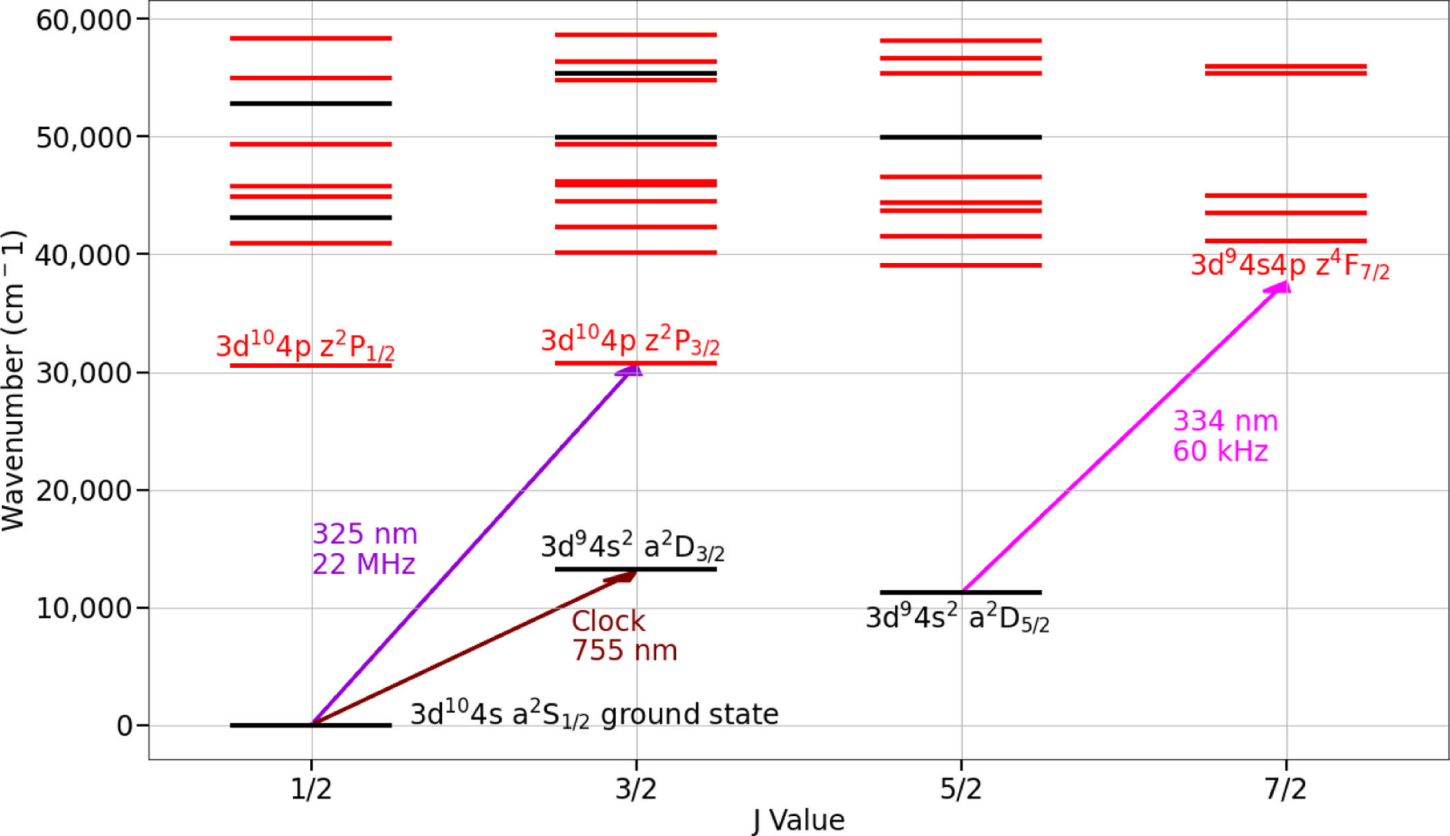
Cu energy level structure with odd (even) parity states shown in black (red). States of interest discussed in the text are indicated. The broad laser cooling line at 325 nm and narrow line at 334 nm ensure efficient cooling. Leaks from the 325 nm cooling transition are limited and readily repumped. The ionization limit is found at 62,317 cm^−1^.

In contrast to Mn, neutral Cu (Z = 29) is characterized by a nearly alkali-like atomic structure (Figure 4). It possesses two stable isotopes with a nuclear spin *I* = 3/2 (^63^Cu and ^65^Cu). Cu’s vapor pressure closely matches that of erbium, ensuring that oven operation is viable. Cu is affordable and readily available. Laser wavelengths are notably more challenging than in Mn but still viable.

As first pointed out in Ref. [26], Cu makes for an interesting clock owing to its small DC Stark sensitivity and altered atomic structure compared to the alkali atoms. Owing to the interplay between a filled d-shell and unfilled s-shell, the ^2^D_*j*_ states drop below the ^2^P_*j*_ states when compared to alkali atoms, offering the prospect for M1/E2 clock transitions (Figure 4); while this introduces complications for laser cooling, it offers a relatively simple atomic structure with a high-accuracy clock transition. In this section, we briefly discuss the prospects for laser cooling as well as the main features of a clock operation.

### 5.1. Laser Cooling

Once more, our discussion on laser cooling is aided by the Kurucz database [45]. Standard MOT operation would proceed from the ground state a^2^S_1/2_ (*F* = 2) to an excited MOT state z^2^P_3/2_ (*F* = 3). Unfortunately, the metastable ^2^D_*j*_ manifold which enables the clock operation leads to sizable branching to metastable states (Table 7). The population in a^2^D_3/2_ is readily repumped through z^2^P_1/2_ (wavelength of 578 nm, linewidth ≈ 300 kHz), where all decay pathways return to the ground state.

Decay during MOT operation will also rapidly populate a^2^D_5/2_ (*F* = 2, 3, 4). Population in the *F* = 2, 3 states may be readily repumped through z^2^P_3/2_, *F* = 2 (wavelength 511 nm, linewidth ≈ 300 kHz) without affecting the MOT operation. The remaining population in *F* = 4 can be handled in two ways. First, it could be repumped through z^2^P_3/2_, *F* = 3 for initial trapping following work performed in neutral barium [49], though this complicates MOT operation owing to dark states. This may be sufficient to initially trap and cool Cu. Alternatively, the narrow line transition indicated in Figure 4 could be used to drive a^2^D_5/2_ (*F* = 4) to z^4^F_7/2_ (*F* = 3), a closed transition which would pump the population in a^2^D_5/2_ (*F* = 4) to other hyperfine states which are repumped. Thus, while the branching ratio of nearly 1:80 found in Cu is undesirable, the relative simplicity of the structure requires a modest number of additional lasers for pumping atoms out of dark states.

We additionally note that optical pumping out of the *F* = 4 state in the a^2^D_5/2_ state is attractive as the same laser can be used to perform narrow-line cooling via z^4^F_7/2_ (*F* = 5). The 60 kHz linewidth of the a^2^D_5/2_ to z^4^F_7/2_ state provides a powerful tool to form a second MOT stage to further cool Cu, as occurs in alkaline-earth-like atoms.

### 5.2. Clock Operation

Clock operation in Cu directly proceeds from the ground state a^2^S_1/2_ (*F* = 1, 2, *m*_*F*_ = 0) to an excited state a^2^D_3/2_ (*F* = 0). We note that the clock wavelength of 755 nm is conveniently realized by optically doubling an s-band laser, meaning a Cu clock would realize a telecom-compatible optical lattice clock. The excited clock state has an estimated lifetime of 4.5 s [45], posing no near-term limits to stability [16].

#### 5.2.1. BBR

Following Section 4.3, we again calculate the BBR shift for the clock transition using the Kurucz database. The fractional BBR shift is found to be 1.5 × 10^−16^, which is in good agreement with Ref. [26]. Once more, we see that utilizing transition metals with transitions between states of the same parity enables clocks with intrinsically lower BBR shifts when compared to the alkaline-earths [17].

#### 5.2.2. AC Stark

One of most challenging aspects of using alkaline-earth-like atoms with suppressed BBR shifts (Cd, Hg, Mg, Be) is the need to use clock lasers and magic wavelengths that are at best in the UV-A spectrum (315–400 nm). Considering the power required for optical trapping, this leads to fragile systems with a low uptime. In contrast, the excited clock state in Cu (a^2^D_3/2_) has strong transitions at both 570 and 578 nm. This guarantees a magic wavelength somewhere between 570 and 578 nm.

Additionally, recall that for the Cu clock transition, the BBR shift is positive, meaning the DC polarizability of the excited clock state is lower than that of the ground state. This ensures zero crossing in differential AC polarizability to the red of the 578 nm wavelength, suggesting that Cu will have a friendly optical magic wavelength.

#### 5.2.3. Hyperfine Structure Induced Second-Order Shifts

Second-order Zeeman shifts for the proposed Cu clock transition were calculated by Dzuba et al. [26]. Owing to the far larger hyperfine splittings for Cu (≈6 GHz for the ground state and 1.7 GHz for the *F* = 0 to *F* = 1 splitting in the excited state), the shifts are strongly reduced compared to Mn. For Cu, the magnitude of the shift is 10 Hz/G^2^, readily reduced to acceptable levels by modest field zeroing.

Just as in Mn, the use of hyperfine states results in a strongly enhanced hyperpolarizability. For Cu, the scale is given by

(22) $$\begin{array}{l} \Delta v_{H}(U)=\frac{{\left(100\text{kHz}\right)}^{2}}{1.7\text{GHz}},\\ \;\approx 6\;\text{Hz}. \end{array}$$

As discussed, the hyperpolarizability may be nulled via detuning from the scalar magic frequency. Compared with Mn, we estimated that this could be readily controlled to the 10^−17^ level of fractional innacuracy before requiring active control. For Cu, this systematic could be pushed well to the low 10^−18^ level, which is compatible with the current Yb and Sr clock-level accuracy.

## 6. Conclusions

In summary, we have proposed alternative types of clock transitions for use in accurate OLCs, all based on transitions between states of the same parity (summarized in Table 8). These transitions are critically built on states that are free of both vector and tensor shifts, following the established protocol in state-of-the-art Yb and Sr OLCs. Via a detailed study of both Mn and Cu, we discuss the advantages and disadvantages of the different architectures, emphasizing how to address each complication. We find that while Mn will require care to achieve the same accuracy as current clocks, it may still achieve a high degree of accuracy while offering applications for continuous clocks. In contrast, Cu with its larger hyperfine splittings may make for a compelling clock with minimal BBR shifts when compared to Sr and Yb clocks. For both Mn and Cu, further study of fourth-order AC Stark shifts to identify magic wavelengths with reduced hyperpolarizabilities would be beneficial.

Enabling additional atoms for clock operation opens the door for a wealth of new research. The suppressed BBR shifts in alternative atoms may avoid the need for a cryogenic operation for state-of-the-art accuracy goals. Some species possess multiple clock transitions, allowing for self-calibration via comparison between different transitions in one platform. Clock transitions between states of the same parity offer enhanced sensitivities to new physics such as the variation of the fine-structure constant [26]. Finally, the J≠0 clock states may be used to explore a wealth of magnetic atomic interactions with the energy resolution of clocks, offering prospects for quantum simulations.

## Figures and Tables

**Table 1.** Examples of atoms with transitions that satisfy the $F=0\to F^{\prime }=0$ clock architecture. Asterisks denote atoms with multiple transitions which fulfill the same criteria.

Atom	Nuclear Spin	Ground State	Excited State	Wavelength (nm)

Co *	7/2	a^4^D_7/2_, *F* = 0	a^2^F_7/2_, *F*^′^ = 0	1509
La *	7/2	a^4^F_7/2_, *F* = 0	a^2^F_7/2_, *F*^′^ = 0	2194
Mn *	5/2	a^6^S_5/2_, *F* = 0	a^6^D_5/2_, *F*^′^ = 0	573

**Table 2.** Examples of atoms which satisfy the J=I, $F=0\to J=1/2^{\prime }$, $F^{\prime }=1,2$, and $m_{F}^{\prime }=0$ clock architecture. Asterisks denote atoms with multiple transitions which fulfill the same criteria. Selected atoms are expected to support long lifetimes.

Atom	Nuclear Spin	Lower State	Excited State	Wavelength (nm)

Cu	3/2	^2^S_1/2_, *F* = 1, 2	^2^D_3/2_, *F*^′^ = 0	755
Sb	5/2	^2^D_5/2_, *F* = 0	^2^P_1/2_, *F*^′^ = 1, 2	1529
As *	3/2	^4^S_3/2_, *F* = 0	^2^P_1/2_, *F*^′^ = 1, 2	550
Ir *	3/2	^4^F_3/2_, *F* = 0	^2^P_1/2_, *F*^′^ = 1, 2	1187
Re *	5/2	a^6^S_5/2_, *F* = 0	a^4^P_1/2_, *F*^′^ = 2	659
Mn *	5/2	a^6^S_5/2_, *F* = 0	a^4^D_1/2_, *F*^′^ = 2	420

**Table 3.** Decay pathways from excited z^6^P_7/2_ state. For each, the wavelength, ground state, and Einstein A coefficient are given. Transitions with A coefficients below 1 Hz are left out. A coefficients are from Ref. [45]. Decay channels offer viable repump transitions for a fast return of MOT-cooled atoms to the Mn ground state.

Wavelength (nm)	Lower State	A (s^−1^)

403.2	a^6^S_5/2_	19.0 × 10^6^
1290	a^6^D_9/2_	4.72 × 10^5^
1330	a^6^D_7/2_	1.24 × 10^5^
1360	a^6^D_5/2_	1.94 × 10^4^

**Table 4.** Decay pathways from excited z^6^F_11/2_ state. For each, the wavelength, ground state, and Einstein A coefficient are given. Transitions with A coefficients below 10 s^−1^ are left out. A coefficients are from Ref. [45].

Wavelength (nm)	Lower State	A (s^−1^)

380.7	a^6^D_9/2_	58.3 × 10^6^
554.1	a^4^G_11/2_	2.47 × 10^3^
554.7	a^4^G_9/2_	1.4 × 10^2^

**Table 5.** Decay pathways from excited y^6^P_7/2_ state for narrow-line cooling. For each, the wavelength, ground state, and Einstein A coefficient are given. Only the dominant pathways are considered. A coefficients are from Ref. [45].

Wavelength (nm)	Lower State	A (s^−1^)

539.6	a^6^S_5/2_	19.4 × 10^3^
6760	a^6^D_9/2_	1.81

**Table 6.** Clock transitions in Mn, all assuming operation from the *F* = 0 a^6^S_5/2_ ground state. Wavelengths and fractional BBR shifts at 300 K are given.

Excited State	Wavelength (nm)	Lifetime (s)	BBR Shift (Fractional)

a^6^D_1/2_	567	1.3	−1.3 × 10^−16^
a^4^D_1/2_	420	725	−1.2 × 10^−16^
a^4^P_1/2_	367	283	4.4 × 10^−16^
b^4^D_1/2_	329	22	4.5 × 10^−16^
a^6^D_5/2_	573	1.4	−2.4 × 10^−16^
a^4^D_5/2_	425	725	−1.9 × 10^−16^

**Table 7.** Decay pathways from excited z^2^P_3/2_ state. For each, the wavelength, ground state, and Einstein A coefficient are given. Transitions with A coefficients below 1 Hz are left out. A coefficients are from Ref. [45].

Wavelength (nm)	Lower State	A (s^−1^)

324.8	a^2^S_1/2_	155 × 10^6^
510.7	a^2^D_5/2_	1.84 × 10^5^
570.2	a^2^D_3/2_	1.34 × 10^5^

**Table 8.** Summary of the proposed clocks with BBR shifts and hyperpolarizability compared to Sr and Yb OLCs. The proposed transition metals demonstrate significantly lower BBR shifts at the cost of enhanced fourth-order AC Stark shifts (hyperpolarizability). The * denotes that Mn has multiple clock transitions.

Atom	Lifetime (s)	BBR Shift (Fractional Magnitude)	Hyperpolarizabilty (Fractional at 100 kHz Trap Depth)

^55^Mn *	1–700	1–5 ×10^−16^	≈10^−13^
^63,65^Cu	4.5	1.5 × 10^−16^	≈10^−14^
^87^Sr	118 [61]	5.5 × 10^−15^ [17]	1 × 10^−18^ [19]
^171^Yb	23 [62]	2.7 × 10^−15^ [17]	6 × 10^−18^ [63]

## Data Availability

Publicly available datasets were analyzed in this study. This data can be found here: https://kurucz.harvard.edu/atoms.html (accessed on 26 February 2024).

## Abbreviations

The following abbreviations are used in this manuscript:

OLC: Optical Lattice Clock
M1: Magnetic Dipole
E2: Electric Quadrupole
MOT: Magneto Optical Trap
